# Fast and nonuniform dynamics of perisaccadic vision in the central fovea

**DOI:** 10.1073/pnas.2101259118

**Published:** 2021-09-08

**Authors:** Janis Intoy, Naghmeh Mostofi, Michele Rucci

**Affiliations:** ^a^Department of Brain & Cognitive Sciences, University of Rochester, Rochester, NY 14627;; ^b^Center for Visual Science, University of Rochester, Rochester, NY 14627;; ^c^Graduate Program for Neuroscience, Boston University, Boston, MA 02215;; ^d^Department of Psychological and Brain Sciences, Boston University, Boston, MA 02215

**Keywords:** saccadic suppression, fovea, microsaccades

## Abstract

Humans use rapid eye movements (saccades) to inspect stimuli with the foveola, the region of the retina where receptors are most densely packed. It is well established that visual sensitivity is generally attenuated during these movements, a phenomenon known as saccadic suppression. This effect is commonly studied with large, often peripheral, stimuli presented during instructed saccades. However, little is known about how saccades modulate the foveola and how the resulting dynamics unfold during natural visual exploration. Here we measured the foveal dynamics of saccadic suppression in a naturalistic high-acuity task, a task designed after primates’ social grooming, which—like most explorations of fine patterns—primarily elicits minute saccades (microsaccades). Leveraging on recent advances in gaze-contingent display control, we were able to systematically map the perisaccadic time course of sensitivity across the foveola. We show that contrast sensitivity is not uniform across this region and that both the extent and dynamics of saccadic suppression vary within the foveola. Suppression is stronger and faster in the most central portion, where sensitivity is generally higher and selectively rebounds at the onset of a new fixation. These results shed light on the modulations experienced by foveal vision during the saccade-fixation cycle and explain some of the benefits of microsaccades.

## Significance

Humans shift their gaze more frequently than their heart beats. These rapid eye movements (saccades) enable high visual acuity by redirecting the tiny high-resolution region of the retina (the foveola). But in doing so, they abruptly sweep the image across receptors, raising questions on how the visual system achieves stable percepts. It is well established that visual sensitivity is transiently attenuated during saccades. However, little is known about the time course of foveal vision despite its disproportionate importance, as technical challenges have so far prevented study of how saccades affect the foveola. Here we show that saccades modulate this region in a nonuniform manner, providing stronger and faster changes at its very center, a locus with higher sensitivity.

Human vision is not uniform across space. While the retina collects information from a broad field, only a minuscule fraction—less than 0.01%—is examined at high resolution. This is the area covered by the foveola, the region void of rods and capillaries, where cones are most densely packed. Because of this organization, rapid eye movements, known as saccades, are necessary to redirect gaze toward the objects of interest, abruptly translating the image across the retina every few hundreds of milliseconds. It is remarkable that the visual system appears unperturbed by these sudden visual transitions and seamlessly integrates fixations into a stable representation of the visual scene.

It has long been observed that visual sensitivity is transiently attenuated around the time of saccades, a phenomenon believed to play a role in perceptually suppressing retinal image motion during eye movements. This effect, known as “saccadic suppression”, consists of the elevation of contrast thresholds to briefly flashed stimuli, which precedes the initiation of the saccade and outlasts it by as much as 100 ms (1–5). Saccadic suppression is typically investigated with stimuli that cover large portions of the visual field, often in the periphery. However, limitations in the precision of stimulus delivery, both spatial and temporal, have so far prevented mapping of the saccade-induced dynamics of visibility within the foveola. Thus, despite the disproportionate importance of foveal vision, little is currently known about its time course around the time of saccades.

Studies on saccadic suppression also commonly focus on large saccades under well-controlled, but artificial, laboratory conditions. However, an examination of the time course of foveal vision needs to take into account that natural execution of high-acuity tasks—the tasks that require foveal vision—normally tends to elicit saccades with very small amplitudes (6). Microsaccades, gaze shifts so small that the attended stimulus remains within the foveola, are the most frequent saccades when examining a distant face (7), threading a needle (8), or reading fine print (9), tasks in which they shift the line of sight with surprising precision. Because of their minute amplitudes, microsaccades pose specific challenges to the mechanisms traditionally held responsible for saccadic suppression (10–19). These movements yield broadly overlapping pre- and postsaccadic images within the fovea, which would appear to provide little masking in visual stimulation (20). They also result in reduced retinal smear (21), as they rotate the eye at much lower speeds than larger saccades, delivering luminance modulations that are well within the range of human temporal sensitivity. Furthermore, it is unknown whether possible corollary discharges associated with microsaccades exert similar effects to those of their larger counterparts (22).

Despite these observations, microsaccades have been found to suppress sensitivity to relatively large test stimuli (23–25). However, the only two studies that specifically examined foveal vision during microsaccades reached diametrically opposite conclusions, with one arguing for a normal reduction in sensitivity (26) and the other for a complete lack of suppression (27), leaving open the question of whether suppression extends to the foveola. While several factors could have been responsible for these discrepant results, two important considerations are worth emphasizing. First, selectively testing foveal dynamics is technically challenging, since the entire foveola is comparable in size to the region of uncertainty in gaze localization resulting from standard eye-tracking methods. Second, the common intuition gained by conceptualizing the visual signals delivered by saccades as uniform—i.e., constant-velocity—translations of the image on the retina (28) does not apply well to microsaccades, whose relatively brief durations and well-defined dynamics yield substantially lower power on the retina than predicted by a uniform translation (29). Thus, even a moderate suppression may be sufficient to prevent visibility of stationary scenes during small saccades.

Recent advances in methods for gaze-contingent display control now enable determination of the line of sight with accuracy sufficient to selectively test a desired foveal region during normal eye movements. Leveraging on these recent advances, here we mapped the perisaccadic dynamics of contrast sensitivity across the foveola during natural visual exploration. We developed a gaze-contingent high-acuity task that resembles primate social grooming, a task that very naturally integrates visual search and detection of brief stimuli and that spontaneously elicits frequent microsaccades, and presented probes at desired retinal locations with high spatial and temporal resolution. Our results show that microsaccades are accompanied by an elevation of visual thresholds at the center of gaze that starts before the initiation of the movement but dissipates very rapidly as the saccade ends. The extent and dynamics of this suppression vary with eccentricity across the foveola, so that a stronger modulation occurs in the most central region, where vision is selectively enhanced after a saccade.

## Results

In a simulated grooming task, observers reported the occurrence of “flea jumps” (the probes), brief changes in the luminance of otherwise dark dots located within the central 2° region of a wide naturalistic noise field. Subjects freely moved their eyes, searching for the locations at which these contrast pulses would occur, while their eye movements were continually recorded.

In reality, unbeknownst to the subject, the probes were activated on the basis of the position and movement of the eye to measure visibility within selected regions of the fovea and at various time lags around saccades. This was possible due to three state-of-the-art components: 1) high-resolution eye tracking achieved via the Dual Purkinje image method (30); 2) accurate gaze localization obtained by means of an iterative gaze-contingent calibration, a procedure that improves accuracy by approximately one order of magnitude over standard methods (6); and 3) real-time control of retinal stimulation, obtained via a custom system for flexible gaze-contingent display control, Eye movement Real-time Integrated System (EyeRIS) (31).

**Fig. 1.**
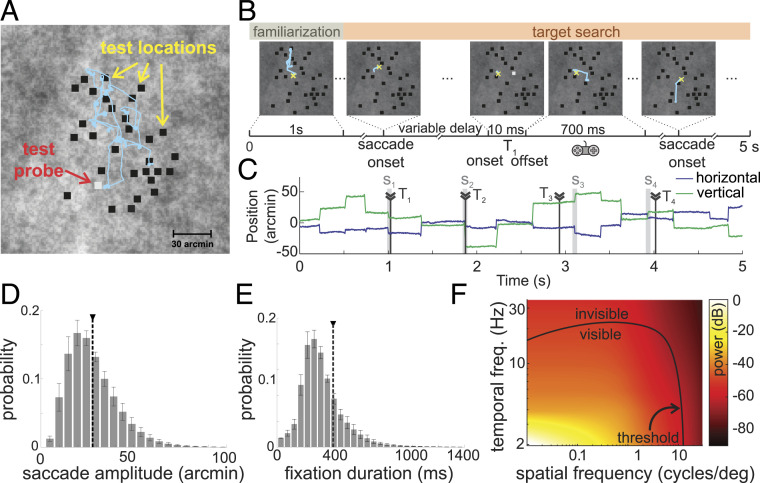
A virtual grooming task. Observers were instructed to search for fleas hiding within the animal’s fur (a naturalistic noise field). (*A*) Thirty dots ($5^{\prime }$ width) were distributed at random test locations across the central 2° region of the display. Subjects were told that a few of these dots were fleas and would distinguish themselves from the remaining dust particles by occasionally jumping (a 10-ms contrast pulse; the probe). The subject’s goal was to “catch” each flea as soon as it jumped by pressing a button on a joypad. (*B*–*D*) Example of a trial. (*B*) Following an initial familiarization period (1 s), the onset of a saccade triggered, with variable delay, a probe ($T_{k}$) at one of the test locations. Both location and timing were selected in real time according to the observer’s eye movements to test performance at various positions in the fovea and lags relative to saccades. The yellow cross and cyan segments represents the center of gaze and eye movements, respectively. (*C*) Gaze position during the course of the trial. Each probe was associated with the closest saccade ($S_{k}$ in C). Only probes with no more than one saccade within a ±200-ms window were selected for data analysis. (*D* and *E*) Characteristics of eye movements. Shown are average distributions of saccade amplitude (*D*) and intersaccadic intervals (*E*) across N = 6 observers. Error bars represent SEM. Vertical dashed lines mark the means of the distributions. (*F*) Power spectrum of the luminance flow delivered to the retina by the recorded saccades. The black line marks contrast sensitivity thresholds in humans [data from Kelly (33)]. The small saccades recorded in this experiment yield visual signals well within the ranges of spatiotemporal sensitivity.

As expected, this high-acuity task resulted in the frequent occurrence of minute saccades. On average, observers executed ∼2.5 saccades per second, almost all of them smaller than 1° (average saccade amplitude and SD across subjects, $28^{\prime }\pm 6^{\prime }$; mean 99th percentile of the amplitude distributions, $68^{\prime }$; Fig. 1*E*). In fact, the majority of saccades (68%) were smaller than $30^{\prime }$, an amplitude range that maintains an initially foveated probe well within the foveola. These tiny gaze shifts occurred at a rate (1.6 microsaccades per second) much higher than those normally encountered in tasks that do not involve high visual acuity (typically <0.2 microsaccades per second) (29), an observation consistent with the notion that microsaccades are normally part of the strategy for examining fine spatial detail (6, 7).

Because of their small amplitudes and stereotypical dynamics (32), the saccades performed in this task resulted in relatively slow changes in visual stimulation. This, combined with the characteristics of the visual scene, which, like natural images, possessed predominant power at low spatial frequencies, resulted in luminance signals to the retina that were well within the range of human temporal sensitivity as measured previously (Fig. 1*F*) (33). Yet, as happens for larger saccades, subjects were not aware of the resulting translations of the images on their retinas—the well-known phenomenon of saccadic omission (3).

**Fig. 2.**
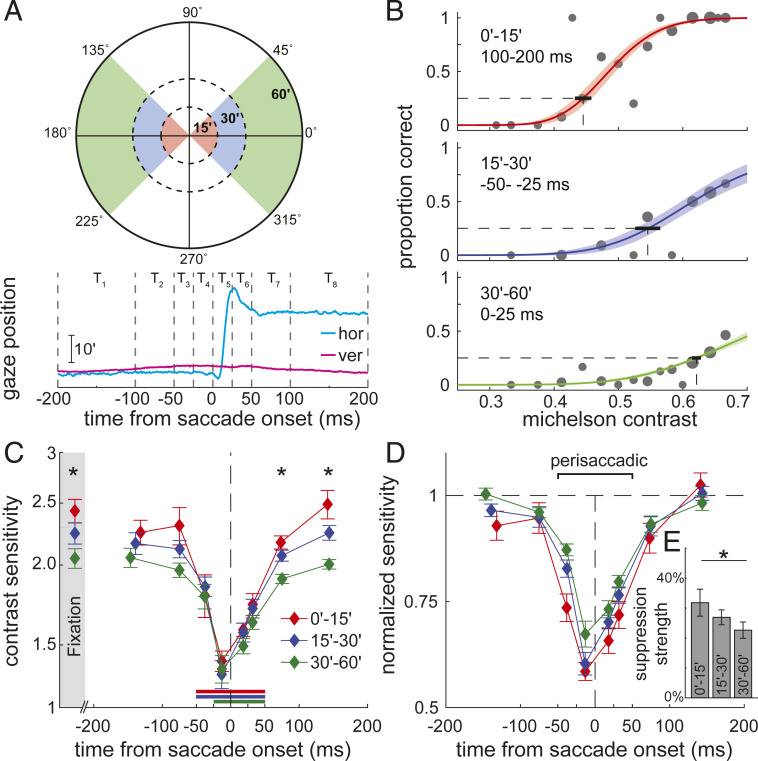
Changes in foveal sensitivity at the time of saccades. (*A*) Contrast sensitivity was measured in 24 spatiotemporal bins around saccades: three distinct regions within the fovea (eccentricity 0 to $15^{\prime }$, 15 to $30^{\prime }$, and 30 to $60^{\prime }$; *Top*); and eight time intervals around a saccade (*Bottom*). (*B*) Contrast sensitivity functions in three spatiotemporal intervals for one observer. Colored lines and shaded regions represent, respectively, the maximum-likelihood fitting and its SEM of a cumulative log-normal function to the data (gray circles; size proportional to the number of samples). The thick horizontal segment represents the SEM of the estimated 25% threshold. (*C*) Dynamics of contrast sensitivity relative to saccade onset. Each line represents mean sensitivity across observers (N=6) in a foveal region. Error bars are SEMs. For comparison, sensitivity measured at fixation, when the probe appeared at saccade lags larger than ±200 ms, is also shown (shaded region). Horizontal bars indicate the intervals in which sensitivity deviated significantly from fixation (P<0.05, post hoc Tukey–Kramer comparisons). ⋆ marks significant differences across foveal regions (P<0.05, one-way ANOVA). (*D*) The same data after normalizing each foveal region by its sensitivity at fixation to highlight differences in dynamics. (*E*) Mean perisaccadic suppression strength across the foveola. Suppression is strongest in the central region (P<0.05, post hoc Tukey–Kramer comparisons).

To quantitatively examine the consequences of saccades on foveal sensitivity, we binned contrast pulses according to their combinations of retinal locations and lags relative to saccade occurrence and separately estimated contrast sensitivity in each spatiotemporal interval (Fig. 2*A*). Fig. 2*B* shows the psychometric functions of contrast sensitivity measured for one subject in three spatiotemporal bins. As these examples show, sensitivity varied considerably not only with the timing of the probe relative to saccades, but also with its position on the retina, reaching, in some instances, low values even at the highest possible contrast.

We first examined sensitivity far from saccades. The data points in the shaded region in Fig. 2*C* represent the average thresholds across observers estimated during fixation, i.e., when no saccade occurred in the surrounding ±200 ms of a probe. Strikingly, despite being separated by just a few arcminutes, the three considered foveal regions exhibited marked differences in sensitivity. Contrast sensitivity was always larger at the very center of gaze and decreased with increasing eccentricity, so that sensitivity in the most central region (the region within $15^{\prime }$) was on average 8% higher than in the range 15 to $30^{\prime }$, which was in turn ∼9% higher than sensitivity in the range 30 to $60^{\prime }$ (one-way ANOVA, F(2,17)=4.8; P=0.02). These measurements reveal how contrast sensitivity varies across the central foveola. They show that, contrary to its anatomical homogeneity, sensitivity is not uniform within this region: Optimal sensitivity is restricted to a very narrow region around the center of gaze during normal fixation.

As the probe approaches the onset of a saccade, drastic changes in visual sensitivity occur. Sensitivity drops sharply from the fixation baseline starting ∼50 ms before the saccade and continues to be affected up to ∼100 ms after the saccade onset, a time at which the saccade has typically already ended (Fig. 2*C*). At all the considered foveal locations, suppression was strongest in the 25-ms interval immediately preceding the saccade, when sensitivity dropped by ∼38% on average. The dynamics of this effect were highly stereotypical across subjects, all of whom individually exhibited a similar and statistically significant attenuation in sensitivity (P<0.05, nonparametric bootstrap; individual subject data in *SI Appendix*, Fig. S1). Thus, the minute saccades performed in our experiment were accompanied by a strong attenuation in sensitivity throughout the foveola, an effect qualitatively similar to the saccadic suppression observed elsewhere in the retina for larger saccades.

While suppression occurred over the entire foveola, the extent and time course of the process differed across foveal regions. All regions ended up with similar visibility levels at the peak of the suppression. However, since sensitivity in distinct regions started from different fixation baselines, the amplitude and speed of the process also varied, so that the change in sensitivity was larger and faster in the most central region of the foveola than at other locations. On average in the 100-ms interval centered at saccade onset, sensitivity was attenuated by 33% in the central region with eccentricity smaller than $15^{\prime }$, whereas it was reduced by only 23% in the $30\text{to}60^{\prime }$ region (P<0.001; post hoc Tukey–Kramer comparison; Fig. 2*E*). Thus, given the similar overall duration of the effect across the foveola, both suppression and recovery were faster at the very center of gaze than at larger eccentricities (Fig. 2*D*).

These results were robust relative to the specific methods for data analysis. Very similar results were obtained by measuring sensitivity to changes in the Weber contrast of the probe relative to its surroundings rather than the Michelson contrast of the probe alone (*SI Appendix*, Fig. S2). Furthermore, differences in foveal dynamics were also reflected in the reaction times of manual responses, which were longer when the probes were less visible (r=−0.50, P<0.01; *SI Appendix*, Fig. S3*A*). At the time of microsaccade onset, the reaction times for probes displayed at the very center of gaze were on average 23% longer than for probes just a few arcminutes away (one-way ANOVA, F(2,16)=8.59, P=0.004; *SI Appendix*, Fig. S3*B*).

**Fig. 3.**
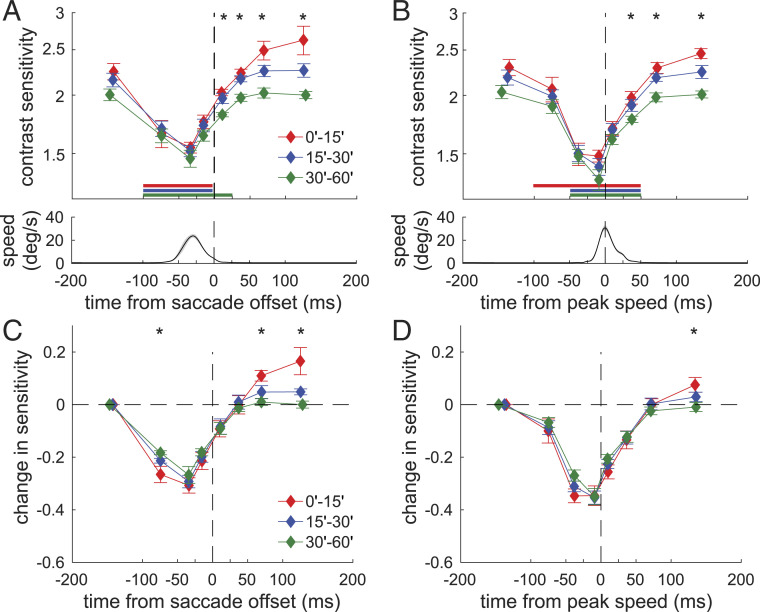
Dynamics of foveal sensitivity. (*A* and *B*, *Top*) Average contrast thresholds are now aligned relative to either (*A*) the time at which the saccade ends or (*B*) the time at which the saccade reaches its peak speed. Graphic conventions are as in Fig. 2*C* with the horizontal bars indicating statistically significant differences relative to fixation (P<0.05, post hoc Tukey–Kramer comparisons). (*A* and *B*, *Bottom*) The mean instantaneous eye speed. (*C* and *D*) The same data normalized relative to the first sample to emphasize differences in dynamics across foveal regions. ⋆ marks significant differences across foveal regions (P<0.05, one-way ANOVA).

To better examine the temporal evolution of saccadic suppression, we recomputed the time course of sensitivity relative to two distinct temporal events, the end of a saccade and the time at which a saccade reaches its peak speed. Visibility recovers extremely rapidly following a saccade. On average across foveal regions, sensitivity has returned to about 90% of its presaccadic value less than 25 ms after the saccade ends and is fully restored within an additional 25 ms (Fig. 3*A*). This happens because suppression largely precedes the actual movement of the eye. Suppression is already recovering by the time a saccade is in midflight and has reached its peak velocity (Fig. 3*B*), an asymmetric temporal evolution that is evident when comparing sensitivity with equal speed of the retina before and after saccade peak speed (*SI Appendix*, Fig. S4).

Normalizing each foveal region by its initial sensitivity further emphasizes the different dynamics occurring at distinct eccentricities. Changes in sensitivity proceed faster in the central region ($<15^{\prime }$), yielding a greater change around 100 to 50 ms before saccade offset than at larger eccentricities (P=0.021; post hoc Tukey–Kramer comparison; Fig. 3*C*). As a consequence, sensitivity is already at its lowest level ∼25 ms earlier in this central region relative to the more peripheral foveola (Fig. 3*D*). These dynamics are little influenced by saccade amplitude. The temporal courses of visibility were almost identical for saccades smaller or larger than $30^{\prime }$, despite the former retaining more power within the range of human temporal sensitivity (*SI Appendix*, Fig. S5).

**Fig. 4.**
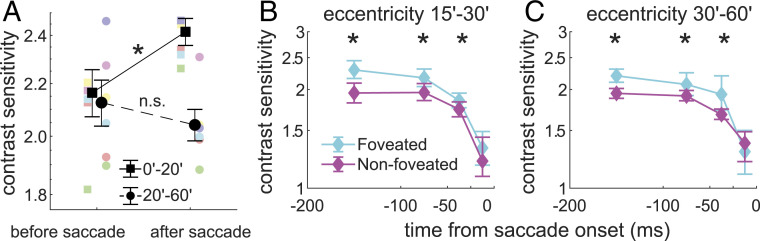
Perisaccadic enhancements in foveal vision. (*A*) Sensitivity is selectively enhanced in the central foveola after a saccade. Black symbols represent average sensitivity measured at least 150 ms before and 50 to 300 ms after a saccade in both the central and peripheral regions of the foveola. Colored symbols are the individual subject data. Error bars represent SEM (^⋆^ P=0.027, paired two-tailed t test). (*B* and *C*) Sensitivity is enhanced in the peripheral foveola before a saccade that lands on the probe (“Foveated”; landing distance $<15^{\prime }$) relative to a saccade that terminates farther away (“Non-foveated”). *B* and *C* show data for the two considered peripheral foveal regions (^⋆^ P<0.05, one-sided nonparametric bootstrap test).

Interestingly, sensitivity rebounds following a saccade, but only in the central portion of the foveola. This effect is clear in the data of Fig. 3*A*, which show that postsaccadic sensitivity continues to increase at the very center of gaze ($<15^{\prime }$) ∼100 ms after a saccade, a time at which sensitivity has already saturated in more eccentric regions (P<0.04; post hoc Tukey–Kramer comparison). To examine in detail this postsaccadic enhancement, we directly compared levels of performance during presaccadic fixation, before suppression started, and in the fixation period that immediately followed a saccade (Fig. 4*A*). In the central foveola at eccentricity smaller than $20^{\prime }$, saccades were followed by higher sensitivity, resulting in an average improvement across observers of 12% (*P* = 0.027; paired two-tailed t test). Such improvement did not occur in the more peripheral region of the foveola ($\geq 20^{\prime }$), where sensitivity decreased slightly following a saccade (*P* = 0.308; paired two-tailed t test), so that these two regions were differently affected by saccades (P=0.001, paired two-tailed t test).

This postsaccadic enhancement is likely the consequence of attention. A similar improvement in sensitivity was also observed before a saccade that landed close to the activated probe, but only outside the central region of the foveola (Fig. 4 *B* and *C*). On average, sensitivity improved by 12% when the planned saccade was toward the probe, suggesting that attention had already moved to this location before shifting gaze. These results further emphasize the different modulations experienced by the distinct portions of the foveola in correspondence of saccades.

The data in Figs. 2–4 show that saccades profoundly modulate foveal vision. To probe into the mechanisms responsible for these effects, we decoupled the visual consequences of saccades from their motor production by passively exposing subjects to the same visual input signals normally resulting from eye movements. In this condition, rather than actively exploring the stimulus, subjects maintained fixation for the entire duration of the trial and reported the activation of the probes in movies that reconstructed the spatiotemporal visual signals previously experienced during normal (i.e., active) execution of the task.

**Fig. 5.**
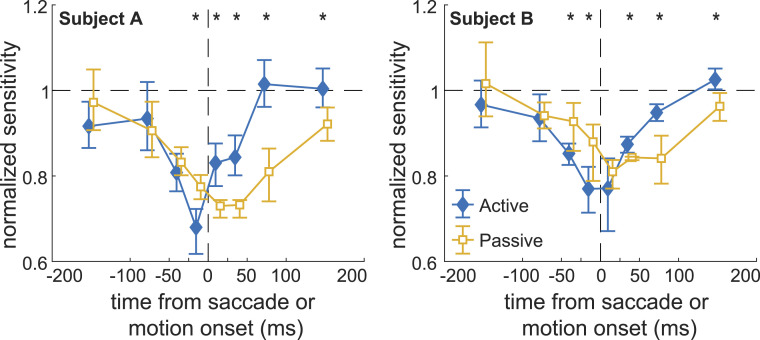
Decoupling the retinal and motor consequences of saccades. Shown are dynamics of contrast sensitivity in correspondence of a saccade (active) and during passive exposure to saccade motion while maintaining fixation (passive). In the latter condition, subjects detected the activation of the probes in movies reconstructing the visual input signals experienced during normal execution of the task. Movies were presented under retinal stabilization to accurately replicate input signals. Data in both conditions are normalized by the sensitivity values measured with probes at least 200 ms away from real or simulated saccades. ⋆ marks significant differences between the two conditions (P<0.05, two-tailed nonparametric bootstrap test).

Passive exposure to saccade motion greatly altered the dynamics of foveal sensitivity (Fig. 5). Performance was impaired in correspondence of the simulated saccades, an effect that may superficially resemble the suppression occurring during real saccades. However, important quantitative differences emerged. With simulated saccades, the reduction in sensitivity was delayed relative to that with real saccades, with peak occurring well after the start of the motion rather than before saccade onset. This reduction was also considerably weaker and persisted for much longer than that observed with real saccades, with sensitivity still impaired 200 ms following motion onset (P<0.02, two-tailed nonparametric bootstrap test). These results point to a combination of retinal and extraretinal mechanisms acting on foveal vision, with an important role played by extraretinal modulations in first suppressing and then enhancing sensitivity respectively before and immediately after a saccade.

## Discussion

Despite its functional importance, vision within the foveola has been critically understudied. Here, we examined the dynamics of foveal vision relative to the minute saccades that naturally emerge during fine spatial exploration. By implementing a naturalistic, yet highly controlled, high-acuity task, we were able to map contrast sensitivity at distinct foveal locations and follow their temporal evolution as eye movements occurred. Our results show that the foveola is accompanied by a general reduction in visual sensitivity in proximity of microsaccades. However, the extent and dynamics of this modulation are not uniform across this region: The attenuation is stronger and faster around the very center of gaze, where sensitivity rapidly rebounds at the end of the movement and remains higher than in the surrounding regions throughout postsaccadic fixation.

By mapping contrast sensitivity and its perisaccadic dynamics across the foveola, our results advance current knowledge of foveal vision in several ways. A first important finding is the observation that during normal intersaccadic fixation, far from the occurrence of saccades, contrast sensitivity is not uniform in the central visual field, but varies by ∼20%. This is a considerable change in such a small region and is consistent with previously reported impairments in discriminating small stimuli located slightly off the preferred retinal locus (34). Previous studies did not measure contrast sensitivity across the fovea, but our results suggest that the way sensitivity declines with increasing eccentricity may have contributed to these impairments. It remains to be determined whether this attenuation in sensitivity originates from attentional modulations that transiently enhance performance at the very center of gaze or more sustained differences in neural processing with eccentricity. But irrespective of the specific mechanisms, our findings further highlight the importance of precisely controlling eye movements in tasks that involve fine spatial judgments, as the preferred retinal locus has a perceptual advantage relative to other foveal regions.

As the time of a saccade approaches, contrast sensitivity to briefly presented stimuli is attenuated throughout the foveola, a deficit that starts ∼50 ms before the onset of the movement and dissipates very rapidly at saccade offset. As it happens outside the fovea for larger saccades, the recovery is faster than the preceding attenuation, leading to greater suppression in the first part of the saccade, before reaching peak speed. Interestingly, differences in modulations occur across foveal regions: Since sensitivity is similarly impaired at the peak of the suppression, but its starting level depends on eccentricity, the amplitude and speed of the process vary across the foveola, so that the change in sensitivity is larger and faster in the most central region. These differences in dynamics are not captured by a simple multiplicative gain, as has been proposed for larger saccades (35), but represent more complex deviations in the shape of the modulations. They persist after normalizing each region by its presaccadic baseline to discount gain differences (Fig. 3). Even after normalization, sensitivity proceeds faster in the most central region, deviates significantly from the other regions before the peak of the suppression (Fig. 3*C*), and levels off ∼25 ms earlier (Fig. 3*D*). In addition, sensitivity rebounds in the central portion of the foveola, an effect that leads to a considerable postsaccadic enhancement at the very center of gaze.

Both retinal and extraretinal processes contribute to these effects. An impairment in detection of the probes was also measured during simulated saccades, when subjects were passively exposed, while maintaining fixation, to the visual input signals normally generated by saccades. This result is not surprising, as the backward masking consequences of saccades have long been discussed in the literature (3, 11, 20), and it is well established that masking also occurs in the foveola (36, 37). However, backward masking was not the sole contributor to foveal suppression, as clear extraretinal influences were visible. Sensitivity dropped faster with real saccades and reached its lowest value before saccade onset, rather than after the onset of motion as with simulated saccades. Sensitivity also recovered much faster with real saccades, an effect similar to that previously observed outside the foveola (11, 38). In addition, enhancements in sensitivity, likely associated with attentional shifts (39, 40), were visible before and after real saccades in the peripheral foveola and at the very center of gaze, respectively.

Qualitatively, these measurements resemble those previously reported outside the foveola, but important quantitative differences occur. Most evident is the overall strength of the effect, which is considerably weaker than the attenuation typically observed with larger saccades (1, 11, 35)—the commonly reported 0.5 to 1 log units suppression. On average across the foveola, sensitivity changed in our experiments by approximately half as much, 0.21 log units, a ∼38% suppression. While multiple factors could have contributed to this effect, it is worth pointing out that this reduction is consistent with the strength of the visual signals resulting from saccades during viewing of stationary scenes. Saccades possess stereotypical dynamics (41) that profoundly affect their luminance modulations, yielding a continuum with the modulations delivered by ocular drift (29)—the incessant intersaccadic motion of the eye (42). On the retina, both saccades and drifts equalize (whiten) the power spectra of natural scenes within a low spatial frequency range. But a trade-off exists between power and bandwidth in this region: The larger a saccade is, the narrower the whitening bandwidth and the higher the power it contains (29). Because of this input reformatting, the power delivered by saccades at low spatial frequencies is considerably less than one may intuitively expect, implying that a strong suppression may not be necessary during natural viewing. Furthermore, this power decreases with decreasing saccade amplitude, providing a possible explanation for the weaker saccadic suppression observed with smaller saccades (43).

Our work differs from previous investigations of visual sensitivity at the time of microsaccades in several important ways. A crucial one is our focus on the foveola, a disproportionally important region of the retina. Most previous examinations used stimuli that covered large visual areas, often excluding the fovea (23–25, 44). Two notable exceptions that specifically focused on foveal vision reached opposite conclusions (26, 27), with the latter reporting lack of suppression during microsaccades. The reduced sensitivity measured in our experiments may help reconcile these previous findings, particularly in the light that—at least for larger movements (45)—suppression tends to be further attenuated for involuntary saccades. Importantly, no previous study has mapped the perisaccadic time course of sensitivity across the fovea, primarily because of the technical challenges inherent in the required spatiotemporal precision of retinal stimulation. These challenges were here overcome by leveraging on recent technological advances in gaze-contingent display (31), which enabled coupling of high-resolution eye tracking with accurate gaze localization and real-time control of stimulus delivery.

This study also differs from previous investigations for its focus on natural visual exploration. Our naturalistic task differs substantially from the simplified stimuli often used by studies of saccade suppression. In these studies, probes are typically easily detectable when saccades do not occur. In contrast, in our experiments, the detection of probe was not always immediate even in the absence of saccades: The average values of sensitivity during fixation were such that it required 50% contrast modulation to reach threshold. Furthermore, studies on saccadic suppression typically focus on instructed saccades when dealing with larger movements and forced fixation when examining microsaccades. Both conditions occur rarely during natural viewing, when subjects typically react to stimuli by redirecting their gaze. Forced fixation, a condition in which observers maintain steady gaze on a marker, also creates a dissociation between the attentional demands of the motor task (the maintenance of fixation) and those of the perceptual task (the detection of a briefly presented stimulus), raising the possibility that disruptions and corrections for fixation mediated by microsaccades may temporarily distract from the visual task, transiently lowering performance.

Here, we focused on the minute saccades that spontaneously emerge during normal examination of fine details. Many of these movements are so small that they maintain the stimulus of interest well within the foveola. Given that subjects could not predict which probe would be activated next, their perseverance with this oculomotor strategy, even after hours of practice, speaks for the importance of this behavior during natural viewing. Previous studies with accurate gaze localization have shown that microsaccades tend to precisely center the stimulus on task-relevant visual details (7–9). Our present results show that this behavior would benefit from at least from two factors, the higher sensitivity around the preferred retinal locus and the sensitivity enhancements that occur around microsaccades. Further benefits may come from the visual transients resulting from saccades, as argued by the proposal of active space–time encoding (42, 46). According to this view, rather than being insensitive to the visual changes caused by saccades, the visual system uses these luminance modulations to encode information during postsaccadic fixation (29, 47–49). These signals likely contribute to the strong neural responses following a saccade (50–52), and their structure is consistent with the sensitivity enhancement measured at low spatial frequencies (53), as well as with its dependence on saccade amplitude (54).

Our results show that contrast sensitivity is not uniform in the central fovea and is modulated by saccades in complex ways. Further work is needed to investigate how these incessant foveal modulations influence oculomotor strategies and how humans actively deal with them to enhance visual performance.

## Materials and Methods

### Subjects.

Data were collected from eight subjects (six females and two males; age range 23 to 33 y). Six subjects participated in the main experiment (Figs. 1–4) and two in the comparison between active and passive exposure (Fig. 5). To ensure high quality of eye tracking and gaze-contingent display control, only emmetropic observers with at least 20/20 acuity, as tested with a standard eye-chart examination, were allowed to participate. With the exception of two of the authors, all observers were naive about the purposes of the experiments and were compensated for their participation. This study was approved by both the Boston University Charles River Campus Institutional Review Board and the Research Subjects Review Board at the University of Rochester. Informed consent was obtained from all participants.

### Apparatus.

Stimuli were displayed on a fast-phosphor calibrated cathode ray tube display (Iiyama HM204DT) at a resolution of 800×600 pixels and a refresh rate of 200 Hz. Observers were maintained at a fixed distance from the display, so that each pixel on the monitor subtended an angle of $∼1.3^{\prime }$. Movements of the head were minimized by means of a head rest and a custom dental-imprint bite bar. Stimuli were viewed monocularly with the right eye while the left eye was patched.

Eye movements were recorded by means of the Dual Purkinje Image (DPI) method, via a generation 6 analog DPI eye tracker (Fourward Technologies). The internal noise of this device has root mean square smaller than 20 s of arc, enabling measurement of eye movements with ∼1 min of arc resolution as assessed by means of an artificial eye. In the active–passive comparison of Fig. 5, stimuli were displayed on a calibrated liquid crystal display (ASUS ROG Swift PG258; 1,920×1,080 resolution and 200-Hz refresh rate), and eye movements were acquired by a custom digital DPI eye tracker with subarcminute resolution (55). Vertical and horizontal eye positions were sampled at 1 kHz, following low-pass filtering (cutoff frequency 500 Hz) of the analog data.

### Stimuli.

Stimuli were designed to loosely replicate the visual input signals experienced by primates while engaged in grooming. They consisted of 30 gray dots (the test locations; each a $5^{\prime }$ dark square at 2.8 cd/m^2^ luminance) simulating “fleas” and “dust particles,” which were randomly distributed within the central 2° region of the display. These objects were displayed over a naturalistic noise-field background, which simulated the “fur” of the animal and covered the entire display, approximately 17° of visual angle. The power spectrum of the background decreased proportionally to the square of the spatial frequency as happens in natural scenes. The average luminance of the display was ∼7 cd/m^2^. An example of the central portion of the stimulus in a trial—the region containing the dots—is shown in Fig. 1*A*. A different noise pattern and a different array of dots were displayed on each trial. Stimuli were rendered in OpenGL and modified in real time according to the observer’s eye movements using EyeRIS, a custom system for gaze-contingent display control (31). This system is designed to guarantee precise timing between changes in the stimulus and oculomotor events (typical delay 7.5 ms) and has been tested extensively and continually refined over the course of a decade.

### Procedure.

Data were collected in separate sessions, each lasting approximately 1 h. Every session started with preliminary procedures to ensure optimal eye tracking and gaze-contingent control. Data were then collected in blocks lasting 10 to 15 min, with breaks between blocks to allow the subject to rest. Every experimental session consisted of five blocks of 40 trials.

Accurate localization of the line of sight was achieved by means of a gaze-contingent two-step calibration already described in previous publications (6, 34). During the first stage of this procedure, subjects completed a standard nine-point calibration by sequentially looking at markers of a 3 × 3 grid. In the second stage, observers used a joypad to finely refine the estimated location of the center of gaze, which was displayed in real time on the monitor. This refinement was also repeated after every trial for the central point of the grid to compensate for possible drifts in the apparatus and/or small head adjustments that may also occur under head immobilization. We have previously shown that this gaze-contingent calibration improves localization of the line of sight by approximately one order of magnitude over standard methods (6).

To measure contrast sensitivity during normal oculomotor activity, we developed a “grooming task,” a high-acuity task designed after primates’ social grooming that naturally incorporates visual search and detection of transient events (Fig. 1*B*). Observers were instructed to search for fleas (dark dots) hidden within the fur of an animal (the noise field). They were told that some of the dots at the test locations were fleas whereas others were dust particles and that the fleas would occasionally reveal themselves by “jumping,” a 10-ms pulse in luminance (the probe) that randomly occurred during the trial. Observers were asked to catch each flea as soon as they saw it jumping by pressing a button on a joypad.

To assess sensitivity at various retinal positions and different times relative to saccades, the eye movements of six observers were continually monitored, and the probes delivered in a gaze-contingent fashion timed to the onset of saccades, as signaled in real time by EyeRIS (estimated instantaneous speed >9°/s). Following detection of a saccade, one of the test locations was selected and the probe activated after a random delay (0 to 400 ms). Selection of the probe location was based on the current location of gaze, to uniformly sample the two quadrants on the retina centered on the horizontal meridian at eccentricities smaller than 1° (Fig. 2*A*). To test one specific retinal location and temporal delay, a saccade could activate only one probe, and each probe was followed by a 700-ms refractory period during which no other contrast pulse occurred. Precise timing of all relevant events was saved by EyeRIS for offline analysis. Subjects were not informed of any of the rules determining the presentation of the probes and remained fully unaware that changes in the display were triggered by their eye movements.

Every trial had a fixed duration of 5 s. It started with the presentation of a new stimulus (a new noise field and pattern of dots) and consisted of two phases: familiarization and search (Fig. 1*B*). No probe was activated during the initial 1-s familiarization phase. Probes were displayed during the search phase and marked as hits if they were followed by a button press within 0.3 to 1 s, an interval chosen based on the typical range of reaction times in this experiment (median 492 ms; 95% confidence interval 311 to 780 ms). In each trial, the change in luminance (the amplitude of the pulse) varied randomly among 20 possible values ranging from 2.8 to 11 cd/m^2^, with the former—the resting luminance level of the probe—corresponding to an intermediate intensity value chosen to minimize phosphor persistence and the latter corresponding to the maximum intensity allowed by the monitor’s settings. Luminance steps varied occasionally across experimental sessions to ensure accurate fitting of the psychometric functions. The number of probes in a trial varied, depending on the numbers of saccades performed by the observer. Subjects were run extensively to estimate contrast sensitivity functions at three retinal eccentricities and eight times relative to saccades. On average, 17,000 probes in 6,000 trials were collected from each observer in ∼30 experimental sessions.

To disentangle the motor and retinal consequences of saccades, an additional condition was introduced in Fig. 5, in which subjects were passively exposed to the same visual signals previously experienced during normal execution of the task. In this condition, subjects reported the activation of the probes in movies that reconstructed the spatiotemporal patterns resulting on the retina from the combinations of the stimuli on the display and the recorded sequences of eye movements. Each movie replicated the visual input of a previously executed normal trial. Subjects maintained fixation for the entire duration of the trial and movies were rendered under retinal stabilization; i.e., they shifted on the display to counteract the ongoing fixational movements of the eye, to replicate retinal stimulation with the greatest possible accuracy.

### Data Analysis.

Recorded oculomotor traces and the events data saved by EyeRIS were examined offline to determine the precise position of each probe on the retina and its timing relative to saccades. Only blink-free trials with optimal, uninterrupted eye tracking were selected for analysis.

We first segmented each trace into complementary periods of saccades and fixations based on the eye speed. Data segments in which the eye displaced by more than $3^{\prime }$ reaching a speed of 3°/s were marked as possible saccades, and their onset and offset were defined as the initial and final times at which the eye speed exceeded and returned to below 2°/s, respectively. Consecutive events closer than 15 ms were merged together, a method that automatically takes care of possible postsaccadic overshoots. Segmentation of the traces was performed automatically and then validated by manual inspection. Saccade amplitude was defined as the modulus of the vector connecting the eye positions at onset and offset.

Each probe was associated with the closest saccade based on temporal proximity. This event was not necessarily the one that triggered the probe during the course of the trial, as other saccades could have occurred closer to the probe, as in the examples S3 and S4 in Fig. 1*C*. Only probes with no more than one saccade within ±200 ms and associated with saccades smaller than 1° were considered in the analyses. To examine visual sensitivity at various visual eccentricities and lags relative to saccades, probes were clustered in 24 spatiotemporal bins (Fig. 2*A*). In space, we mapped performance in three eccentricity ranges in the ±45° circular sectors centered on the horizontal median: 0 to $15^{\prime }$, 15 to $30^{\prime }$, and 30 to $60^{\prime }$. In time, we examined the evolution of contrast sensitivity at eight intervals around selected saccadic events: saccade onset, offset, and peak velocity.

Psychometric functions of contrast sensitivity were independently evaluated in each of these bins via a maximum-likelihood procedure (examples in Fig. 2*B*). A cumulative log-normal function was fitted to the performance data measured at various contrasts by means of the Psignifit Matlab toolbox (56). The contrast sensitivity values reported in Figs. 2–5 are the inverse of the Michelson contrast,

$$C=\frac{I-I_{0}}{I+I_{0}}=\frac{\Delta I}{2I_{0}+\Delta I},$$

where $I_{0}$ represents the baseline intensity of the probe (∼3 cd/m^2^) and ΔI is the change in luminance of the pulse ($I=I_{0}+\Delta I$). Virtually identical results were obtained by measuring changes in Weber contrast of the probe relative to its surroundings (*SI Appendix*, Fig. S2). To follow the dynamics of the low visibility measured around the time of saccades, we summarized performance by the contrast thresholds yielding 25% correct detection. For each subject and spatiotemporal bin, variability in the estimated threshold was assessed by nonparametric bootstrap over 1,000 random samples of the probes (error bars in *SI Appendix*, Fig. S1). Subjects were run extensively to collect a sufficient amount of data for a reliable estimation of contrast thresholds in all bins. On average, 10,000 probes were used to construct the spatiotemporal map of contrast sensitivity for each individual, corresponding to ∼65% of the total number of probes.

The spectral density map in Fig. 1*F* was obtained by reconstructing the luminance signals delivered by the recorded saccades and estimating their power spectra. The spectral analysis was conducted using an approach that allows high spatial resolution, as previously described in the literature (29), and then averaged across subjects. The lines mapping the range of visibility are typical contrast thresholds taken from classical measurements of human sensitivity in the absence of retinal image motion (33).

In the analysis of Fig. 4 *B* and *C*, we compared presaccadic sensitivity when the probe location was targeted by the saccade (the saccade landed within $15^{\prime }$ of probe) and when the saccade landed farther away. To reach sufficient numbers of trials in which the saccade relocated gaze on the activated probe, we estimated thresholds via bootstrap on the ensemble of trials pooled across subjects.

## Supplementary Material

Supplementary File

## Data Availability

The contrast thresholds data and matlab scripts used to produce the figures in the main text have been deposited into the Open Science Framework repository (https://osf.io/jqg59/).
