# Supplementary material for: Fast and nonuniform dynamics of perisaccadic vision in the central fovea
Source: Proc Natl Acad Sci U S A. 2021 Sep 8;118(37):e2101259118. doi: 10.1073/pnas.2101259118 (PMC8449317; doi:10.1073/pnas.2101259118)
Supplement: Supplementary File [file pnas.2101259118.sapp.pdf]

PNAS
www.pnas.org

Supplementary Information for

Fast and nonuniform dynamics of perisaccadic vision in the central fovea

Janis Intoy, Naghmeh Mostofi, and Michele Rucci

Correspondence: jintoy@bu.edu (J.I.) and mrucci@ur.rochester.edu (M.R.)

This PDF file includes:

Figs. S1 to S5

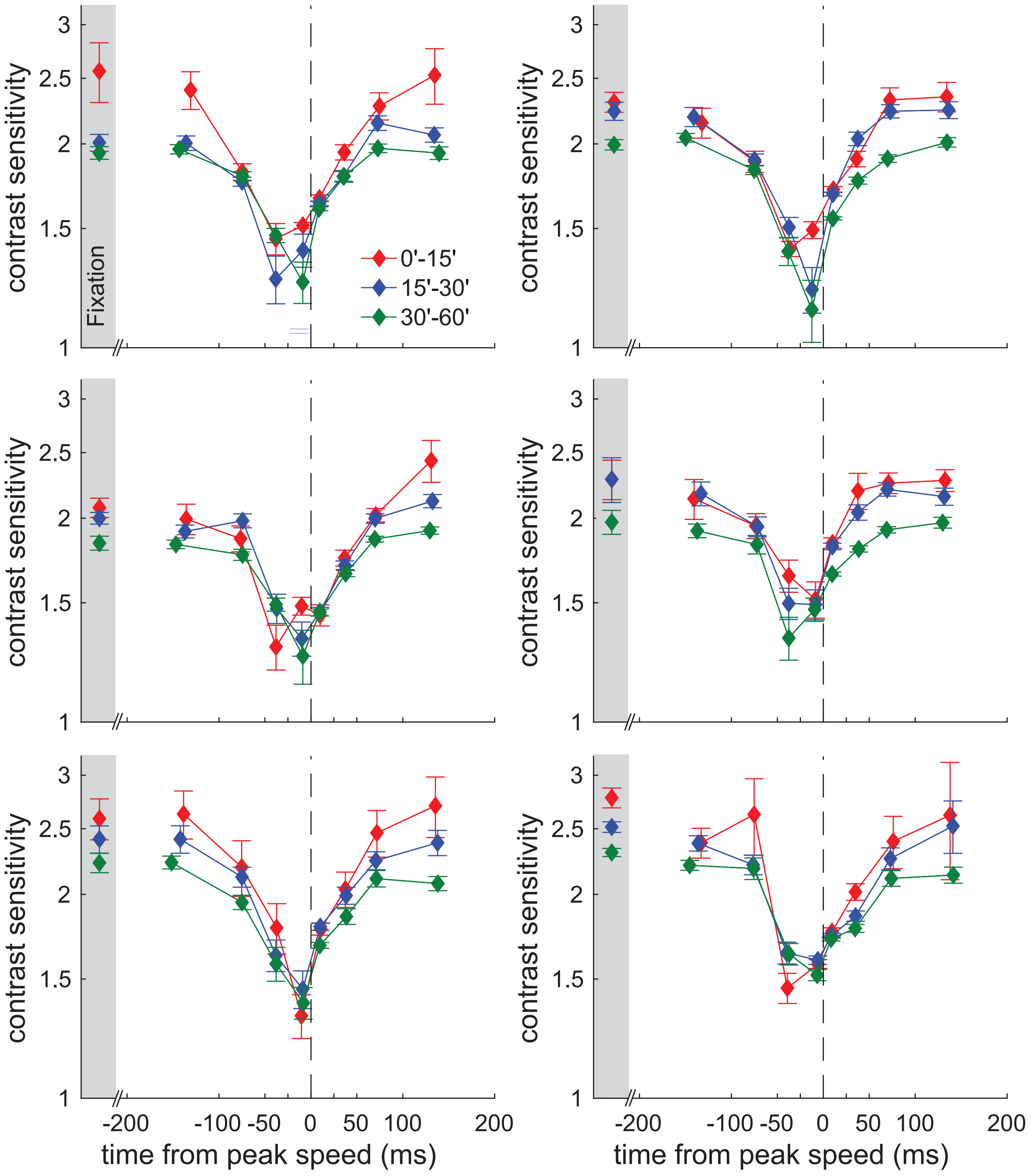

Fig. S1. Individual subjects' data. Dynamics of contrast sensitivity relative to saccade peak speed. The data from the N=6 subjects are shown on separate panels. Graphics conventions are as in Fig. 2C. Error bars represent SEMs of the 25% contrast thresholds obtained from 1000 bootstrap repetitions.

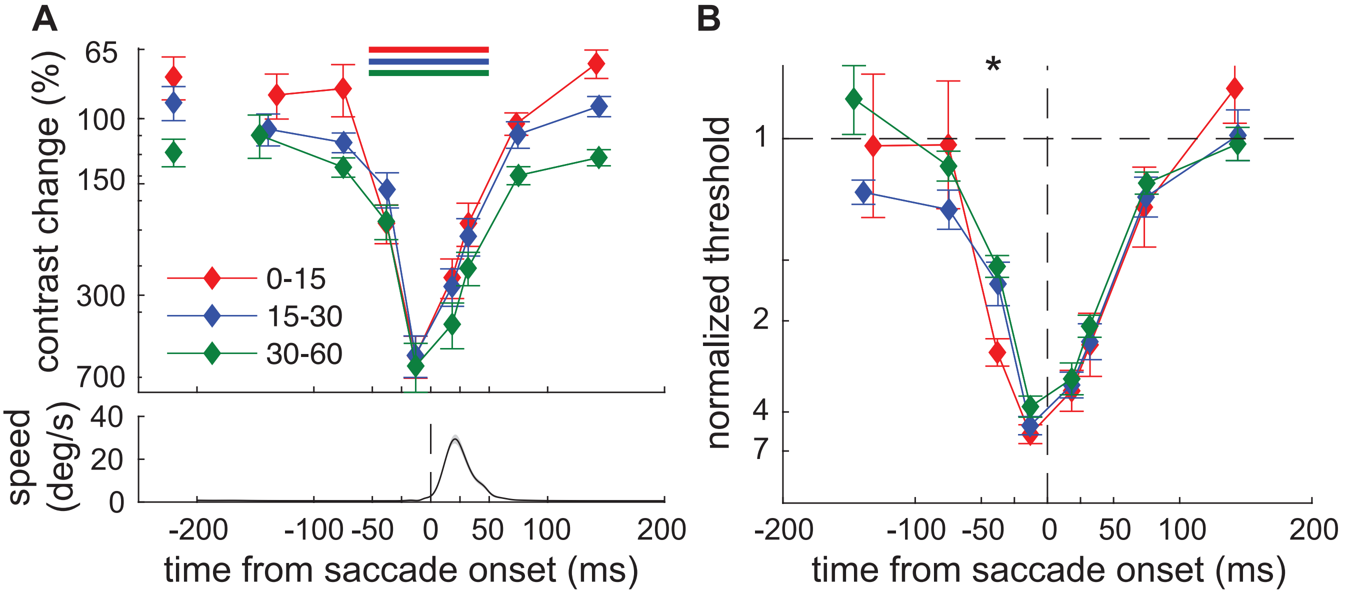

Fig. S2. Robustness of results. Highly similar results were obtained by measuring sensitivity to changes in the Weber contrast of the probe relative to its neighborhood rather than the Michelson contrast of the probe alone. (A) Dynamics of sensitivity to Weber contrast changes relative to the time of saccade onset. Data represent 25% performance thresholds $(C_1 - C_0)/C_0$ averaged across subjects, where C_0 and C_1 are, respectively, the Weber contrasts of the dot before and after activating the probe relative to the average luminance of the display within the surrounding $10'$-radius circle. Horizontal bars indicate the intervals in which sensitivity differed significantly from the sample $\sim$150 ms before saccade onset (P <0.05, post hoc Tukey-Kramer comparisons). (B) The same data normalized by the threshold measured at fixation. Values greater than 1 indicate visual suppression. Stars mark the intervals with statistically significant differences across eccentricities (one-way ANOVA, F(2, 17) =7.96; P =0.004).

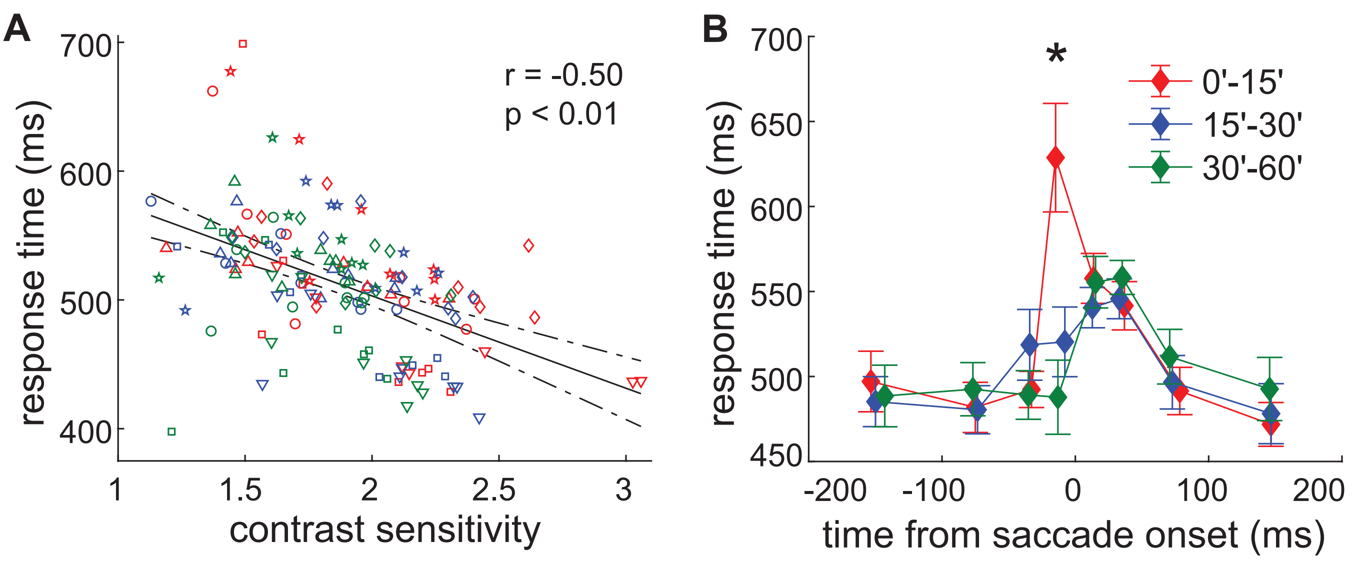

Fig. S3. Manual response times. (A) Mean delay in reporting the probe as a function of contrast sensitivity. Each data point represents the mean delay and sensitivity in one spatiotemporal bin (eccentricity marked by color as in B) for an individual observer (marked by different symbols). (B) Response time as a function of the probe's time of appearance relative to saccade onset. The three curves are the average response times across observers at the three tested eccentricities. Error bars represent SEM. Spatiotemporal bins are here aligned relative saccade onset ($\star$ one-way ANOVA, P <0.05).

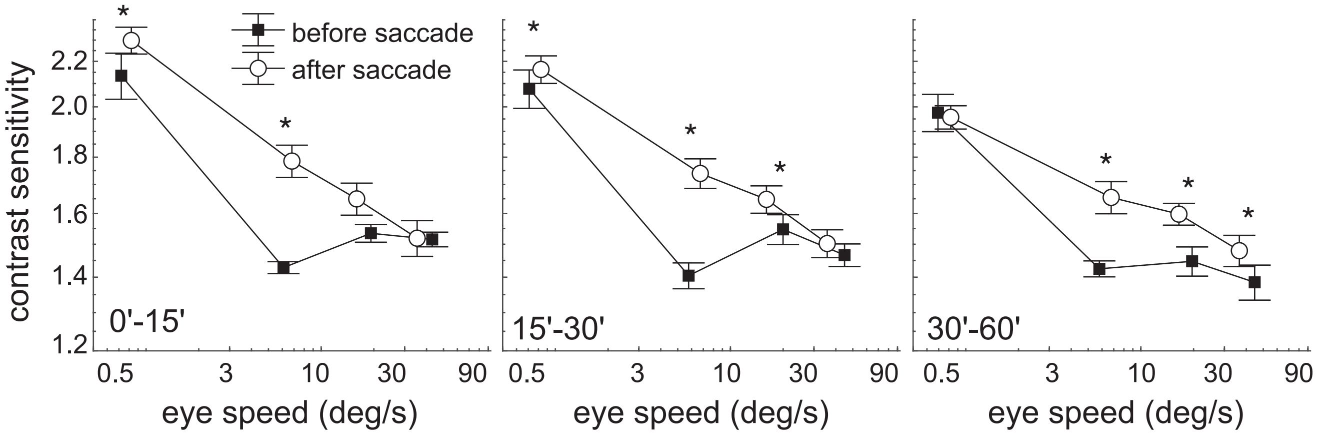

Fig. S4. Asymmetric dynamics of saccadic suppression in the foveola. Contrast sensitivity as a function of eye speed before and after the saccade peak velocity. Sensitivity is generally lower in the initial part of the saccade (an 8% attenuation on average; $P = 0.002$, paired two-tailed t test). In this period, sensitivity is already fully attenuated at low speeds, whereas it declines monotonically with ocular speed later during the saccade. The three panels show data for the three considered ranges of eccentricities. $\star$ marks significant differences before and after the saccade peak speed ($P < 0.05$; paired two-tailed t test).

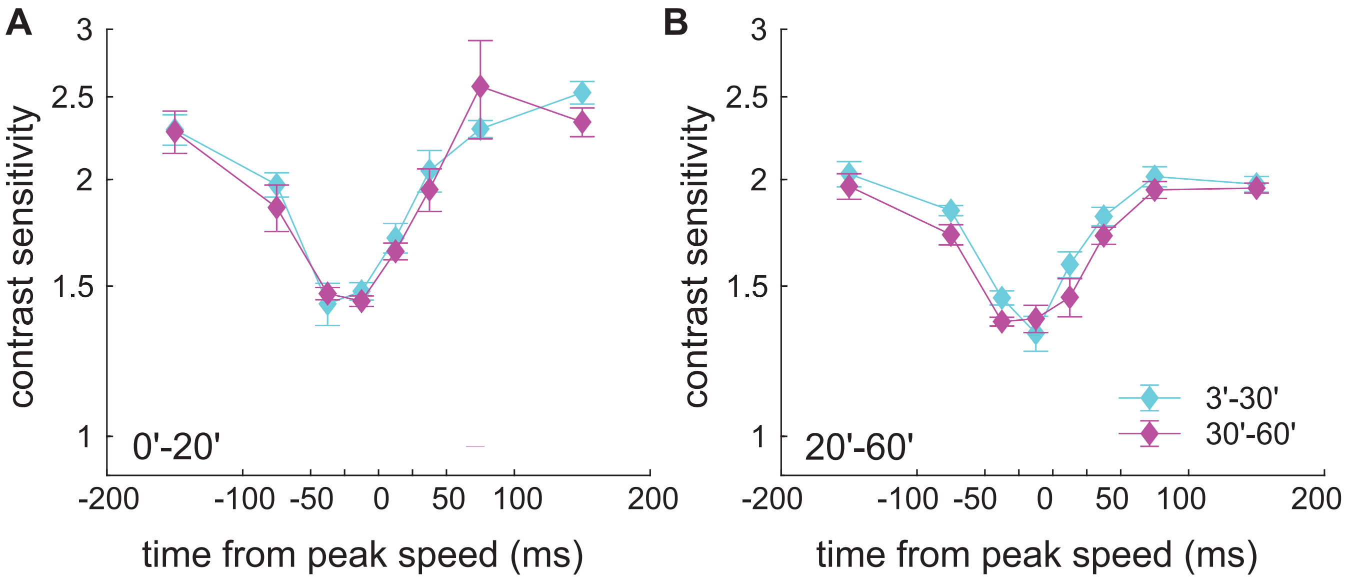

Fig. S5. Foveal dynamics is little affected by saccade amplitude. Changes in contrast sensitivity around saccades in distinct amplitude ranges: $<30'$ and $30'$ to $60'$. To counteract the loss in statistical power resulting from partitioning the saccade samples, we accumulated more data from each individual observer by comparing performance in two—rather than three—foveal regions: (A) the central region with eccentricity smaller than $20'$; and (B) the more peripheral region at $20'$ to $60'$. Data represent averages across observers (N=6) aligned relative to the time of peak speed. Error bars are SEMs.
